# Band to Band Tunneling at the Zinc Oxide (ZnO) and Lead Selenide (PbSe) Quantum Dot Contact; Interfacial Charge Transfer at a ZnO/PbSe/ZnO Probe Device

**DOI:** 10.3390/ma12142289

**Published:** 2019-07-17

**Authors:** Minkyong Kim, Chang-Yeol Han, Heesun Yang, Byoungnam Park

**Affiliations:** Department of Materials Science and Engineering, Hongik University 72-1, Sangsu-dong, Mapo-gu, Seoul 04066, Korea

**Keywords:** ZnO, PbSe, quantum dot, field effect transistor, localized states, charge transfer

## Abstract

We provide a comprehensive understanding of interfacial charge transfer at the lead selenide (PbSe) quantum dot (QD)/zinc oxide (ZnO) interface, proposing band to band tunneling process as a charge transfer mechanism in which initial hopping of carriers from ZnO to PbSe QDs is independent of temperature. Using the transmission line method (TLM) in a ZnO/PbSe/ZnO geometry device, we measured the ZnO/PbSe electrical contact resistance, a measure of charge transfer efficiency. Fabrication of a highly conductive ZnO film through Al doping allows for the formation of ZnO source and drain electrodes, replacing conventional metal electrodes. We found that band to band tunneling at the PbSe QD/ZnO interface governs charge transfer based on temperature-independent PbSe QD/ZnO contact resistance. In contrast, the PbSe QD channel sheet resistance decreased as the temperature increased, indicating thermally activated transport process in the PbSe QD film. These results demonstrate that, at the ZnO/PbSe QD interface, temperature-independent tunneling process initiates carrier injection followed by thermally activated carrier hopping, determining the electrical contact resistance.

## 1. Introduction

Efficient interfacial charge transfer is of central importance in assembling highly efficient display and energy storage/conversion devices [1,2,3,4,5]. Modern electronic devices consist of stacked layers in which the single component layers are incorporated for efficient charge transfer and transport during device operation. For optimization of device performance, band gap engineering has been extensively carried out for various materials [6,7,8,9]. Particularly, quantum dot (QD) materials have attracted much attention because of their excellent electronic tunability through quantum confinement effect. QD size-dependent optoelectronic properties including energy band gap and carrier mobility arising from quantum confinement effect have accelerated applications to light emitting diodes (LEDs) and energy harvesting research fields including photovoltaics (PVs) [10,11,12].

Among a class of QDs, IV-VI semiconductor lead salts (PbS, PbSe, and PbTe) have been used as an emitting layer in near infrared QD LEDs as well as a light sensitizer in highly efficient PVs [13,14,15,16]. Lead salts feature high dielectric constants and low effective masses, exhibiting strong quantum confinement effects resulting from large Bohr radii [17]. PbSe QDs have, particularly, shown high carrier mobility and decent tunability of energy band gap through ligand and band gap engineering, demonstrating to be a versatile building block for nanostructured QD devices [18,19,20].

QD films have been incorporated as an emission layer and a light sensitizer in LEDs and PVs, respectively. The combination of QDs with an electron transport layer (ETL) was demonstrated to be very effective in injecting and extracting carriers for device operation [16,21,22]. In particular, ZnO has been adopted in many devices as electron transport and/or electron accepting layers. Interfacial and compositional engineering to tune energy level alignment between QDs and ZnO has been carried out for device optimization. In LEDs, for example, unbalanced carrier injection has been considered as a major origin of the efficiency roll-off resulting from non-radiative Auger recombination [23,24]. Enhanced external quantum efficiency was achieved with charge carrier balance through modifying the surface of the ETL, which controls the interfacial charge transfer rate between the QD emission layer and the ZnO layer [25,26]. Non-luminescent localized sites at the QD/ZnO interface were also passivated through adjusting energy band alignment at the interface, suppressing non-radiative recombination process [23].

For highly efficient PVs, interfacial charge transfer/transport associated with the localized states in the ZnO has been focused. Hoye et al. reported improvement in the open circuit voltage through band gap tuning of ZnO using Mg doping (Zn_1−x_Mg_x_O) in which the precise position of the conduction band tail in the ZnO increased the open circuit voltage from 408 mV to 608 mV [27]. Importantly, the acceptor states close to the bottom of the band tail were found to cause a loss in the open circuit voltage, limiting the power conversion efficiency of colloidal QD PVs. Timp et al. investigated energy level alignment between PbSe QD and ZnO using ultra-violet photoelectron spectroscopy [28]. The position of the conduction band minimum (CBM) energy level of the PbSe QDs was found to be independent of the QD size ascribable to the electronic interactions at the ZnO/PbSe interface which possesses high density of surface states. In contrast, size-dependent open-circuit voltage in PVs containing surface-treated PbSe QDs was observed [29].

As demonstrated in the previous studies of LEDs and PVs mentioned above, a significant complication at the ZnO/QD interface arising from interfacial localized states between ZnO and QD makes it difficult to develop a systematic optimization process. Energy band offset engineering has been extensively carried out, but defect-induced charge injection and transport have turned out to be crucial in determining interfacial carrier injection and transport, which is closely related to power conversion efficiency and external quantum efficiency in PVs and LEDs, respectively [1,2,3,4]. Mainly, a high surface to volume ratio of QDs limits correlation energy level alignment as well as electronic structures associated with localized states to charge transfer efficiency. Charge injection rate depending on complicating interfacial electrostatic conditions and energy band offset are required to be quantified into the magnitude of the electrical contact resistance, enabling interpretation into the charge transfer efficiency. For a more direct study, we configured an electrical contact probe system in which the ZnO/PbSe QD interface is embedded in a ZnO/PbSe/ZnO geometry device. This strategy allows us to investigate complicating interfacial electronic properties through electrical contact resistance measurements, enabling interfacial defect engineering through which an effective contact barrier can be measured and tuned. As mentioned earlier, we chose the combination of ZnO and PbSe QDs because the combination ensures a model functional interface system in the near-infrared PVs and LEDs in which charge transfer is unavoidable for device operation. Particularly, PbSe QDs possess abundant ligand chemistry and facile tuning of optoelectronic properties through strong quantum confinement due to its large Bohr radius, which is potentially promising for a variety of functional interface formations.

In our previous study, electrical analysis of the ZnO/PbSe contact properties revealed that the QD ligand and size-dependent interfacial traps determine the charge transfer efficiency as well as interfacial charge transport [30]. Carrier mobility of PbSe in close proximity to the ZnO electrode depends on the choice of QD size and ligand, and the consequent interfacial charge transport was demonstrated to govern the contact resistance, a measure of charge transfer rate [30].

Here, we report the detailed charge transfer mechanism dominating the ZnO/PbSe interfacial contact properties through simultaneous probing of charge transport and transfer, based on temperature-dependent contact and channel sheet resistance measurements. The detailed transfer mechanism associated with band to band tunneling through localized states is discussed along with initial hopping of carriers from ZnO into PbSe QD, and QD size-dependent injection energy barrier, providing a comprehensive understanding of interfacial charge transfer at the QD and ZnO.

## 2. Materials and Methods

To synthesize PbSe QDs, we mixed PbO (4 mmol) and oleic acid (10 mmol) in 1-octadecene (ODE) producing a precursor solution with [Pb] = 0.3 M and a molar Pb:oleic acid ratio of 1:2.5 followed by degassing at 160 °C for 1 h under nitrogen. We prepared Se in trioctylphosphine (TOP) in a glovebox and then diphenylphospine (DPP) (4.5 mM) was blended with the TOP-Se solution (12 mL, 1 M), producing PbSe QDs after injection. By adjusting temperature, reaction time, and molar Pb:oleic acid ratio the size of PbSe QDs was tuned. After reaction, the solution was quenched in a water bath. To prepare 1,2-ethanedithiol (EDT)-treated PbSe QD (~3 nm) films, we spin-coated PbSe QD films onto the patterned ZnO contacts followed by EDT-treatment in which PbSe QD films were immersed in a solution of EDT in acetonitrile (ACN, 0.01 M) for 10 min. EDT-treatment was finalized with ACN rinsing. The detailed synthesis and PbSe QD film preparation conditions are described elsewhere [30].

To fabricate ZnO electrodes, we prepared ZnO films using sputtering. Al_2_O_3_ target (3 wt%) was used for Al-doping. Highly conductive ZnO source and drain electrodes were formed by annealing at 200 °C for 2 h in air followed by annealing at 500 °C in a nitrogen box. We patterned the sputtered ZnO films such that PbSe QDs could be deposited between the ZnO electrodes as the electrical channel. For fabrication of a ZnO/PbSe/ZnO geometry electrical probe device, we patterned the channel and the contact regions through which a ZnO film sputtered on an SiO_2_ substrate is selectively removed for PbSe QD deposition while remaining ZnO regions become the source and drain electrodes. For ZnO patterning, we covered the ZnO surface with positive photoresist followed by UV exposure through a patterned mask in which the source and drain regions are closed and the channel region is open. After UV exposure, the photoresist above the channel region is eliminated while the photoresist above the electrode regions remains during a develop process. Dipping the patterned device in a dilute HCl causes the channel region of ZnO to be etched forming an electrical channel between the ZnO electrodes protected by the photoresist. ZnO/PbSe/ZnO devices are finalized through spin-coating (1500 rpm for 30 s) of PbSe solution in hexane (10 mg/mL) on the ZnO patterned devices producing a 90 nm thick PbSe QD film. The thickness of the PbSe QD film was measured using a profilometer. To increase the conductivity of PbSe QD films, we replaced oleic acid ligands by EDT [30]. For electrical measurements, we mounted samples in a cryostat vacuum chamber, enabling temperature-dependent contact and channel sheet resistance measurements up to 77 K.

## 3. Results and Discussion

**Figure 1:**
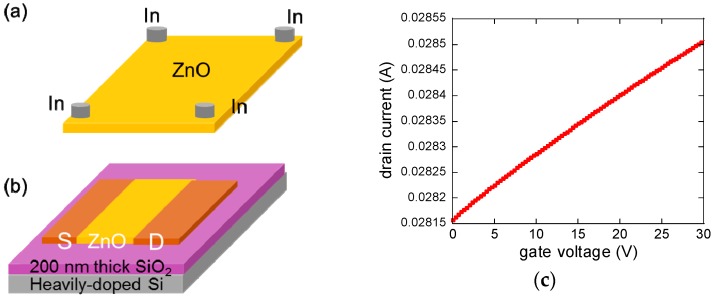
Electrical properties of Al-doped ZnO films. (**a**) Schematic of a Hall effect measurement device. Indium contacts are spaced by ~5 mm. Applied current is 2 mA. (**b**) ZnO field effect transistor (FET) devices to measure the FET mobility. The thickness of ZnO is ~90 nm. The source and drain electrodes (Au/Ti) are 80 nm thick. (**c**) Transfer characteristic curve of a ZnO FET. The drain current was fixed at 5 V.

The electrical properties of highly conductive Al-doped ZnO were measured using Hall and field effect transistor (FET) configurations. We measured the Hall mobility of as-deposited ZnO film using the van der Pauw geometry in Figure 1a and the electrical resistivity was calculated to be ~0.004 Ω·cm. The magnitude of the applied current was 2 mA, while the measured voltage at four indium contacts were hundreds of milli volt. The majority carrier type from the Hall measurement was *n*-type and the Hall mobility was 103 cm^2^/Vs. We also fabricated a bottom-contact ZnO FET structure as displayed in Figure 1b in which a highly doped Si served as a gate electrode. A 200-nm thick SiO_2_ layer was used as a gate dielectric. From the transfer characteristic curve in Figure 1c, the FET mobility was estimated to be 44 cm^2^/Vs. A substantial difference between the FET mobility and the Hall mobility is attributed to a high contact resistance due to a large energy band offset between the Fermi energy of Au (5.2 eV) and the conduction band minimum of ZnO (4.3 eV) in the FET geometry device. The other possible reason is that the FET method probes the ZnO close to the gate dielectric, wherein interfacial carrier scattering can reduce the mobility of ZnO while the Hall mobility reflects the ZnO bulk properties over the entire thickness. The structural properties of a ZnO film were measured in the supporting information Figure S1 using X-ray diffraction (XRD) wherein diffraction peaks corresponding to 002 and 004 were observed.

**Figure 2:**
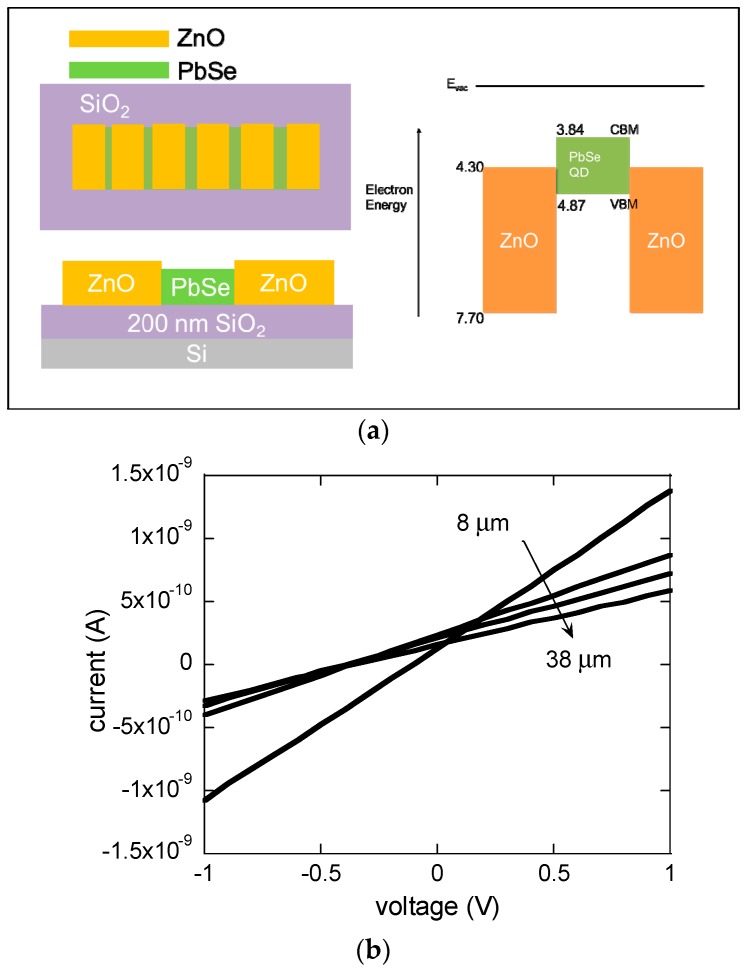

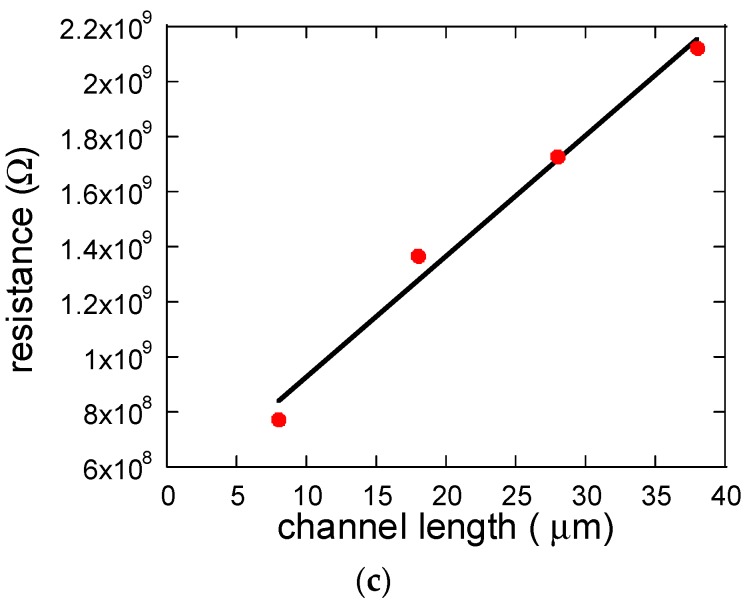
(**a**) Schematic diagram of a ZnO/PbSe/ZnO test platform device. ZnO (70 nm) source and drain electrodes and 3 nm PbSe quantum dot (QD) films (90 nm) are patterned onto a 200 nm thick SiO_2_ substrate. The channel length between ZnO source and drain electrodes varies between ~6 and ~40 μm. The channel width is 3 mm. (**b**) Current–voltage curves at different channel lengths of 8, 18, 28 and 38 μm at 293 K. (**c**) Plot of total resistance as a function of channel length for calculation of contact and channel sheet resistances at 293 K.

To probe the quantum confinement effect in the synthesized PbSe QDs, we measured size-dependent optical absorption and the results are shown in the supporting information Figures S2 and S3. The estimated PbSe QD size from XRD (Rigaku, Seoul, Korea, Debye-Scherrer method) was consistent with the size from transmission electron microscopy (TEM, HITACHI, Seoul, Korea) images of PbSe QDs as shown in the Supplementary Materials Figure S3. Size-dependent quantum confinement effect of PbSe QDs (~3 and ~4 nm) features prominent 1st exciton absorption peaks at around 1032 and 1360 nm, respectively, which are consistent with TEM images and XRD data. From the estimation of the conduction band minimum (CBM) and the valence band maximum (VBM) in our previous study [30], the energy band diagram of the ZnO/PbSe (~3 nm)/ZnO arrangement is predicted, as shown in Figure 2a.

The test platform device structure to probe ZnO/PbSe contact properties is displayed in Figure 2a in which the channel length varies from ~8 to ~38 µm. As the channel length increased, the conductance decreased, as seen in Figure 2b. The contact resistance (*R_c_*) in the ZnO/PbSe/ZnO structure was measured using transmission line method (TLM) in which the electrical resistance of the channel (PbSe) resistor was systematically varied by increasing the PbSe channel length. The total resistance (*R_tot_*) is extracted from the current–voltage curves at different channel lengths in Figure 2b. The contact resistance at the source and drain electrodes, and the channel resistance (*R_ch_*) are related to the channel sheet resistance (*R_s_*), the channel length (*L*) and the channel width (*Z*) as given in the following equation:

(1) $$R_{tot}=R_{ch}+R_{c}=R_{s}\frac{L}{Z}+R_{c}$$

The contact resistance is calculated by extrapolating the total resistance to the zero channel length in the *R_tot_* vs. *L* curve. The channel sheet resistance is proportional to the slope of the *R_tot_* vs. *L* curve. From the TLM analysis of Figure 2c, the contact and channel sheet resistances were 4.87 × 10^8^ Ω and 1.32 × 10^11^ Ω_sq_, respectively.

**Figure 3:**
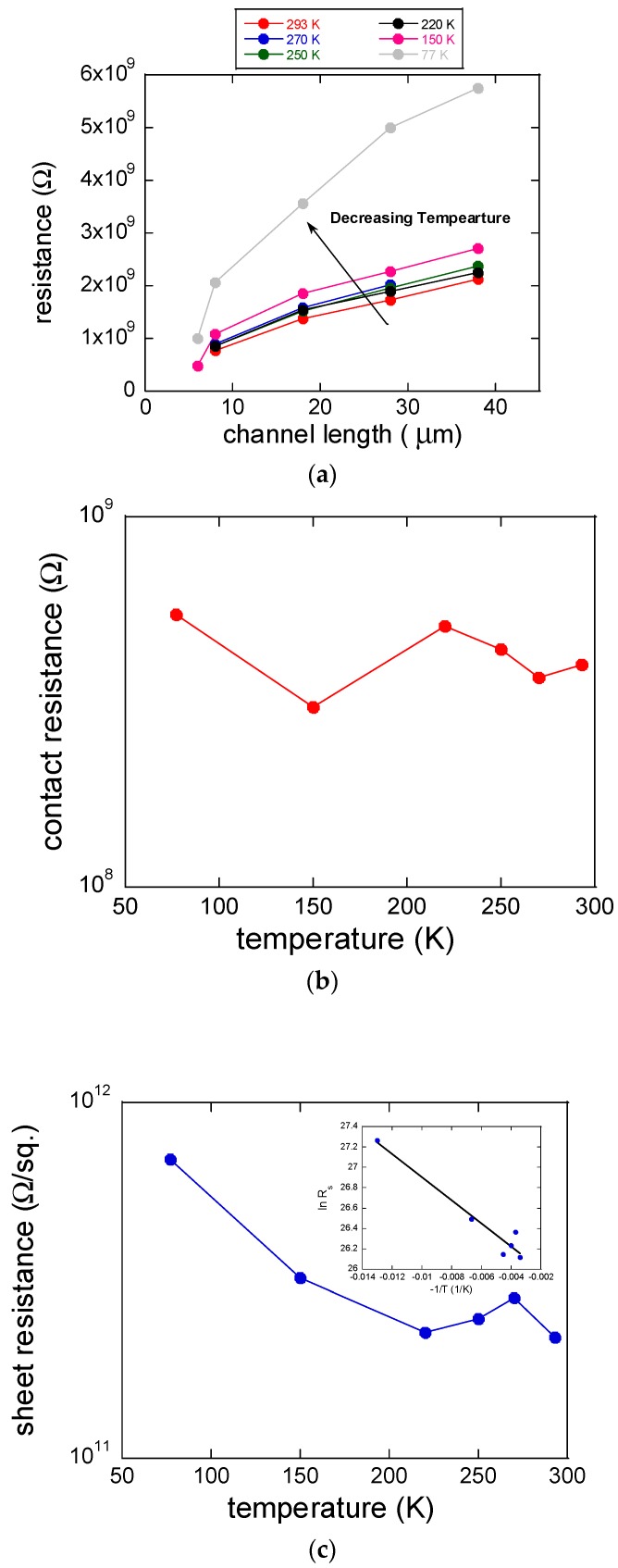
(**a**) Plot of total resistance as a function of channel length (6, 8, 18, 28, and 38 μm) at different temperature ranging between 77 and 293 K. (**b**) Plot of ZnO/PbSe QD contact resistance as a function of temperature. (**c**) Plot of channel sheet resistance as a function of temperature. The inset shows a plot of ln *R_s_* vs. −1*/T* to calculate thermal activation energy based on Arrhenius equation.

To elaborate on the charge transfer mechanism at the ZnO/PbSe interface, we calculated the contact resistance and the channel sheet resistance at different temperatures. Figure 3a shows the plots of the total resistance as a function of channel length at a temperature range between 77 and 293 K. Temperature dependence of the contact and channel properties extracted from Figure 3a are shown in Figure 3b,c. Importantly, the contact resistance change was not significant at the temperature range, while the channel sheet resistance decreased as the temperature increased, indicating that charge transport in a PbSe QD film is thermally-activated with an activation energy of ~10 meV as derived from the curve in the inset of Figure 3c. To calculate the activation energy, *E_A_*, for charge transport based on Arrhenius equation (*R_s_ = R*_0_*exp*(*−E_A_/k_B_T*), *k_B_*: Boltzmann constant), the channel sheet resistance, *R_s_*, was plotted as a function of −1/*T*. The sheet resistance was measured from the relation, $R_{tot}=R_{ch}+R_{c}=R_{s}\frac{L}{Z}+R_{c}$, in which the slope of the *R_tot_-L* plot is multiplied by the channel width (*Z*). For calculation of activation energy, the slope in the plot (ln*R_s_* vs. −1/*T*) is multiplied by *k_B_*.

**Figure 4:**
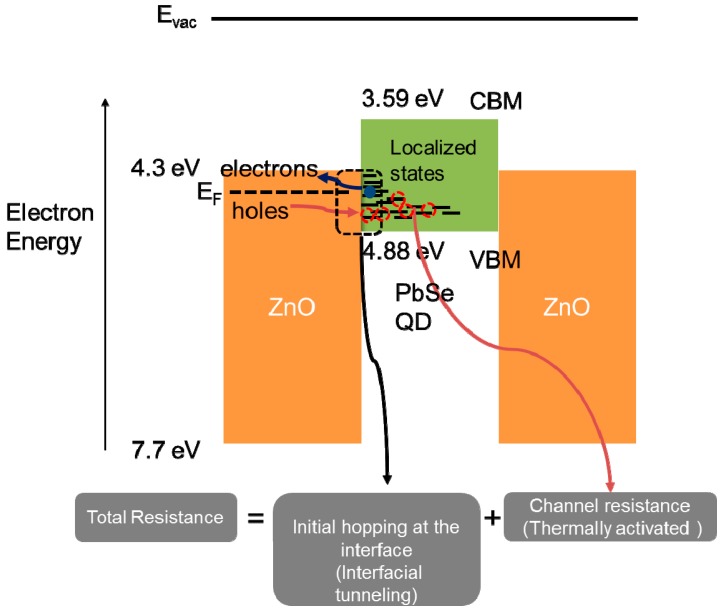
A schematic of ZnO/PbSe (3 nm) charge transfer mechanism in which carrier injection process is dominated by band to band tunneling through localized states in the PbSe QD film.

**Figure 5:**
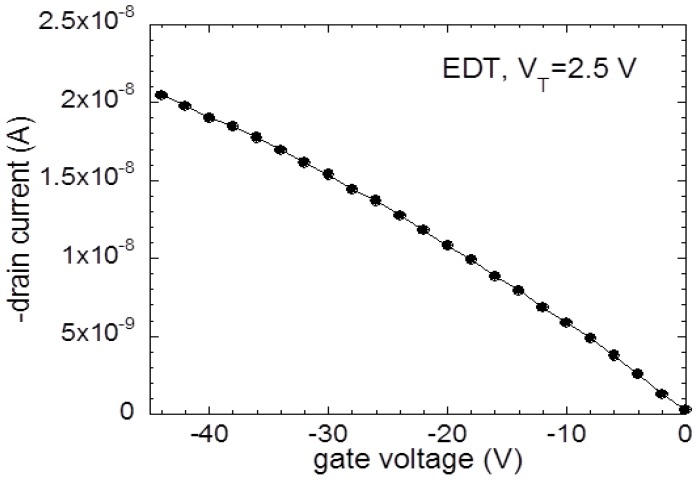
Transfer characteristic curve for a PbSe FET treated with 1,2-ethanedithiol (EDT). The drain voltage was fixed at −5 V. The channel length and width are 80 μm and 2 mm, respectively. The PbSe QD film thickness ranges between ~50 and ~80 nm.

From the results of the contact and channel sheet resistance measurements, it is found that two consecutive processes govern the charge transfer rate at the ZnO/PbSe contact, as described in Figure 4. Holes are injected through a tunneling process from ZnO to PbSe which is independent of temperature. Through FET measurements in Figure 5, we found that the majority carrier in the PbSe QD film is holes, inferring that hole transfer is initiated through a tunneling process from ZnO to PbSe. It is important to note that the threshold voltage for a *p*-channel EDT-treated PbSe FET was 2.5 V. This emphasizes that mobile hole carriers are available in the absence of the gate electric field, enabling initial hopping of holes from the ZnO to the PbSe through the localized interfacial states via temperature-independent tunneling process. The injected holes are transported through thermal activation in the channel region as evidenced by temperature-dependent channel sheet resistance measurements, in which the sheet resistance of the PbSe QD film decreased with temperature increasing.

The band to band tunneling mechanism at the ZnO/PbSe interface, followed by thermally activated hole transport validates carrier injection and extraction between ZnO and PbSe film, even in the presence of a large energy band offset for carrier injection or extraction at the ZnO/PbSe interface. Disordered PbSe QD films in contact with ZnO through ligand exchange favors the formation of localized states which serve as carrier tunneling path at the interface. In our previous study, indeed, higher trap density induced by ligand exchange increased the contact resistance as well as the channel sheet resistance [30].

The presence of the band to band mechanism provides a complementary view in elaborating on the charge transfer rate at the ZnO/PbSe interface. It is important to emphasize that charge transfer rate at the PbSe/ZnO partly depends on the position of the localized states relative to the VBM level of the PbSe. In our previous study, we found that the contact resistance depends on the QD size, concluding that, as the QD size increases the contact resistance decreases due to lower carrier injection barrier as well as enhanced electrical mobility [30]. Charge injection barrier across the ZnO/PbSe interface was derived from field-enhanced thermionic emission model from which the ratio of the contact resistance between large (~4 nm) and small (~3 nm) diameter PbSe QD films was estimated to be ~0.1 eV. This value is, indeed, comparable to the injection barrier inferred from the size-dependent PbSe QD energy levels estimated from the first excitonic absorption peaks [31]. As the PbSe NC size increases, the localized states in the PbSe as well as the VBM level of the PbSe is positioned closer to the conduction band edge of the ZnO, lowering hole injection barrier. This result clarifies that the initial hopping rate of carriers from ZnO to PbSe is partially determined by the position of the localized states relative to the ZnO which depends on the size of QD.

## 4. Conclusions

In summary, we fabricated a ZnO/PbSe/ZnO test platform device to probe interfacial contact properties. We observed that the ZnO/PbSe contact resistance change was not substantial with temperature, clarifying that at the interface temperature-independent tunneling process dominates carrier injection, while thermally activated carrier hopping occurs in the channel region away from the ZnO/PbSe interface. In other words, two consecutive processes determine the interfacial contact properties. Initial hopping of carriers from ZnO to PbSe is governed by band to band tunneling mechanism and thermally activated transport process dominates channel transport. This work demonstrates that band to band transport through tunneling between localized states is involved in determining charge transfer efficiency, emphasizing that defect engineering at the functional interface can be applied for device optimization for energy and display applications.

## Supplementary Materials

The following are available online at https://www.mdpi.com/1996-1944/12/14/2289/s1, Figure S1: (a) θ–2θ and (b) ω-rocking scans for an Al-doped ZnO film, Figure S2: Optical absorbance spectra of PbSe nanocrystals after washing. Bottom curve has an excitonic peak at 1032 nm corresponding to a diameter of ~3 nm, top curve has an excitonic peak at 1360 nm have a diameter of ~4 nm, Figure S3: (a) TEM image of PbSe QDs (~3 nm). (b) Optical absorbance spectra of ~4 and ~5 nm PbSe nanocrystals and size measurement from Debye-Scherrer method.
