# Supplementary material for: Band to Band Tunneling at the Zinc Oxide (ZnO) and Lead Selenide (PbSe) Quantum Dot Contact; Interfacial Charge Transfer at a ZnO/PbSe/ZnO Probe Device
Source: Materials (Basel). 2019 Jul 17;12(14):2289. doi: 10.3390/ma12142289 (PMC6678409; doi:10.3390/ma12142289)
Supplement: Supplementary file 1 [file materials-12-02289-s001.pdf]

materials

Supplementary Materials: Band to Band Tunneling at the Zinc Oxide (ZnO) and Lead Selenide (PbSe) Quantum Dot Contact; Interfacial Charge Transfer at a ZnO/PbSe/ZnO Probe Device

Minkyong Kim, Chang-Yeol Han, Heesun Yang * and Byoungnam Park *

Structural Properties of ZnO Films

ZnO films were grown on 200 nm thick SiO_2/Si substrate using RF magnetron sputtering. The film was grown in Ar atmosphere at 400 °C growth temperature by using 2-inch ZnO single ceramic target doped with 3 wt% Al_2O_3. The base pressure prior to deposition was lower than 1×10^{-6} Torr and working pressure of 4×10^{-3} Torr was maintained at an Ar gas flow rate of 20 sccm. The deposition rate of 3 nm/min was obtained at RF power of 40 W. The crystalline quality of Al-doped ZnO films was evaluated by X-ray diffraction (Smartlab, Rigaku Co.) of θ–2θ and ω-rocking scans using a Cu Kα radiation. X-ray diffraction data showed diffraction peaks at 2θ = 34.17°, 2θ = 72.12° are corresponding to 002, and 004 plane of Al:ZnO, respectively. Full width of half maximum (FWHM) of ZnO 002 was 4.3°.

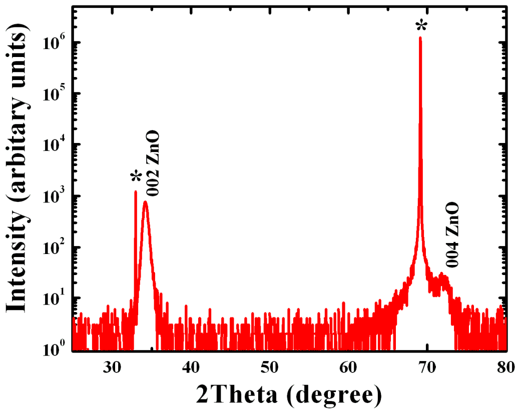

(a)

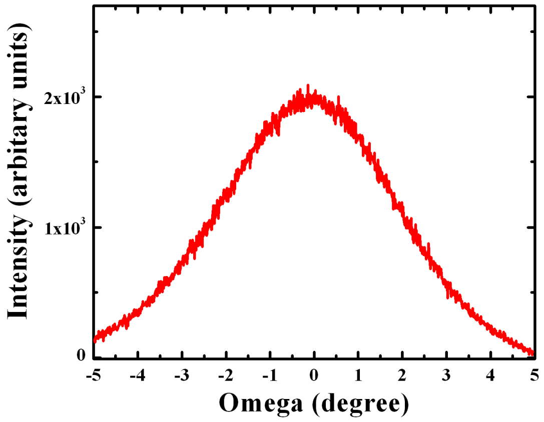

(b)

Figure S1. (**a**) θ–2θ and (**b**) ω-rocking scans for an Al-doped ZnO film.

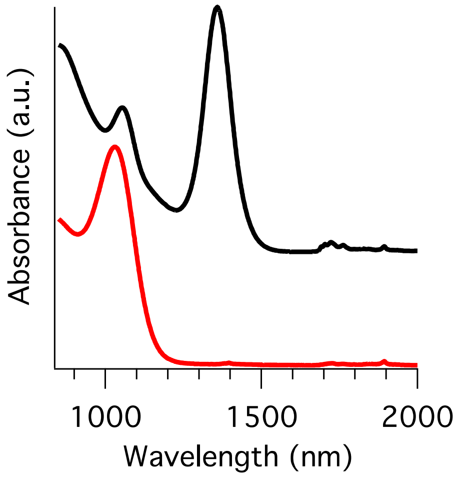

Figure S2. Optical absorbance spectra of PbSe nanocrystals after washing. Bottom curve has an excitonic peak at 1032 nm corresponding to a diameter of ~3 nm, top curve has an excitonic peak at 1360 nm have a diameter of ~4 nm.

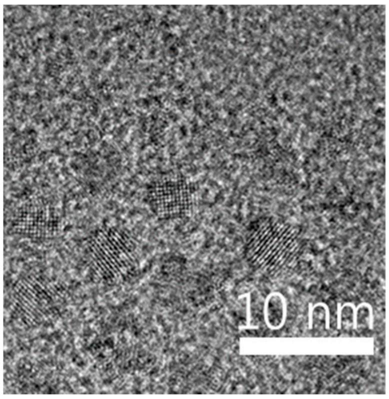

(**a**)

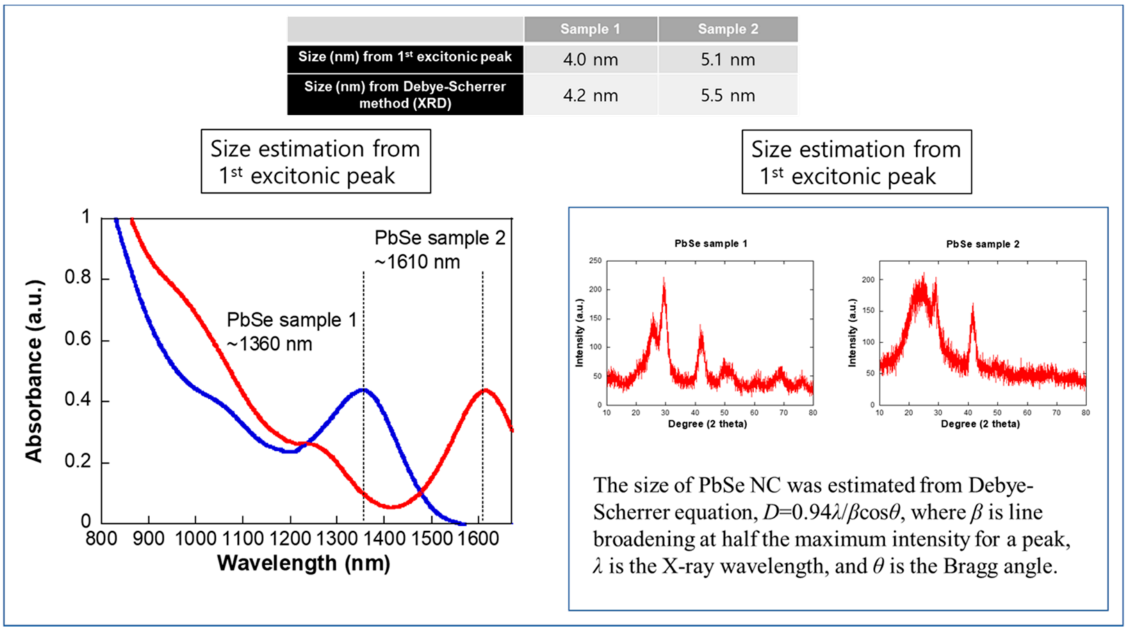

(**b**)

Figure S3. (**a**) TEM image of PbSe QDs (~3 nm). (**b**) Optical absorbance spectra of ~4 and ~5 nm PbSe nanocrystals and size measurement from Debye-Scherrer method.

In extracting the CBM and VBM levels, we estimated size from the 1st excitonic peak. To validate this method we made 4 and 5 nm sample to confirm. As shown in the above figure, the size from excitonic peak position and the Debye Scherrer method was very well matched.
